# The basic circuit of the IC: tectothalamic neurons with different patterns of synaptic organization send different messages to the thalamus

**DOI:** 10.3389/fncir.2012.00048

**Published:** 2012-07-26

**Authors:** Tetsufumi Ito, Douglas L. Oliver

**Affiliations:** ^1^Department of Anatomy, Faculty of Medical Sciences, University of FukuiEiheiji, Japan; ^2^Research and Education Program for Life Science, University of FukuiFukui, Japan; ^3^Department of Neuroscience, University of Connecticut Health Center, FarmingtonCT, USA

**Keywords:** GABA, glutamate, local circuit, inferior colliculus

## Abstract

The inferior colliculus (IC) in the midbrain of the auditory system uses a unique basic circuit to organize the inputs from virtually all of the lower auditory brainstem and transmit this information to the medial geniculate body (MGB) in the thalamus. Here, we review the basic circuit of the IC, the neuronal types, the organization of their inputs and outputs. We specifically discuss the large GABAergic (LG) neurons and how they differ from the small GABAergic (SG) and the more numerous glutamatergic neurons. The somata and dendrites of LG neurons are identified by axosomatic glutamatergic synapses that are lacking in the other cell types and exclusively contain the glutamate transporter VGLUT2. Although LG neurons are most numerous in the central nucleus of the IC (ICC), an analysis of their distribution suggests that they are not specifically associated with one set of ascending inputs. The inputs to ICC may be organized into functional zones with different subsets of brainstem inputs, but each zone may contain the same three neuron types. However, the sources of VGLUT2 axosomatic terminals on the LG neuron are not known. Neurons in the dorsal cochlear nucleus, superior olivary complex, intermediate nucleus of the lateral lemniscus, and IC itself that express the gene for VGLUT2 only are the likely origin of the dense VGLUT2 axosomatic terminals on LG tectothalamic neurons. The IC is unique since LG neurons are GABAergic tectothalamic neurons in addition to the numerous glutamatergic tectothalamic neurons. SG neurons evidently target other auditory structures. The basic circuit of the IC and the LG neurons in particular, has implications for the transmission of information about sound through the midbrain to the MGB.

## Introduction

The inferior colliculus (IC) is a hub of the auditory nervous system. It receives inputs from virtually all brainstem auditory nuclei and the auditory cortex, and it sends axons to the medial geniculate body (MGB). The basic neural circuit of the IC is defined by the different types of neurons, their inputs and their outputs, and they are the topic of our review.

A basic circuit for the IC should be composed of common components. Within each subdivisions of the IC, i.e., central nucleus (ICC), and the dorsal and lateral cortices (DC and LC), there may be common cellular components. However, the neuron types may be distributed in unequal proportions within each subdivision. Likewise, the inputs from specific afferent pathways may not terminate uniformly (Oliver et al., 2003; Cant and Benson, 2006; Loftus et al., 2010). There may be, however, patterns of input common to all of IC. Finally, the outputs of IC may come from neurons with different dendritic morphology, i.e., disc-shaped or stellate, but the differences in the dendritic morphology may not predict the pattern of projection (Oliver, 1984; Oliver et al., 1991). There may be other aspects of neurons that are better correlated with their axon targeting. These would be the components of the basic IC circuit.

## Neuron types in the IC

Neurons in the IC have been defined on the basis of dendritic morphology, neurotransmitter synthesis, and synaptic organization. Morphologically, two basic types of IC neurons have been identified in Golgi preparations. Disc-shaped (flat) neurons extend their dendrites parallel to the fibrodendritic laminae, while stellate (less flat) cells have spherical or elliptical dendritic fields that often cross the borders of fibrodendritic laminae (Oliver and Morest, 1984; Malmierca et al., 1993). This morphology may be important to shaping the inputs of the IC neuron and relevant to the frequency bandwidth of the response to sound. There are subtypes of neurons of different somatic and perikaryal size (see also Paloff et al., 1989). Paloff also has divided IC neurons into spiny and non-spiny varieties (Paloff et al., 1992), but there has been little subsequent study of spine morphology or function in the IC.

The IC contains GABAegic and non-GABAergic neurons (Roberts and Ribak, 1987; Oliver et al., 1994; Merchan et al., 2005). Roughly 20–25% of the IC neurons are GABAergic (Oliver et al., 1994; Merchan et al., 2005). The remaining 75% are glutamatergic based on their expression of one of the vesicular glutamate transporters (VGLUT), specifically VGLUT2 (Ito et al., 2011). Disc-shaped and stellate cells can be GABAegic or non-GABAergic (Oliver et al., 1994). Thus, the dendritic morphology does not predict the neurotransmitter synthesis of the neuron. As we will see below, it also fails to predict the axonal target of the neuron. Therefore, a single morphological or neurochemical signature is insufficient to identify the neuron type in the IC. However, recent findings on the synaptic organization of GABAegic and non-GABAergic neurons appear to better distinguish different neuronal types that also can be identified with specific outputs, and this may clarify the neural circuit in which they are embedded.

### Identification of axosomatic inputs on large GABAergic neurons

Excitatory axosomatic inputs are seen on only a subpopulation of neurons, and this distinguishes them from other neuronal types in the IC. Glutamate, the main excitatory neurotransmitter in CNS, is loaded into synaptic vesicles by VGLUT (Takamori et al., 2000). Because these proteins are presynaptic, they are excellent markers for glutamatergic presynaptic axonal terminals. Three subtypes of VGLUT have been found (VGLUT1, VGLUT2, and VGLUT3) (Takamori, 2006). Of these, only VGLUT1 and VGLUT2 were found axonal terminals of the adult rat IC (Altschuler et al., 2008; Ito et al., 2009). Both VGLUT1 and VGLUT2 terminals are abundant throughout the neuropil of the IC and contact many dendrites. However, Altschuler and colleagues discovered that only VGLUT2-immunopositive terminals made dense contacts on the somata and proximal dendrites of a subpopulation of large IC neurons, while VGLUT1-positive terminals were only seen in the neuropil on dendrites (Altschuler et al., 2008).

**Figure 1:**
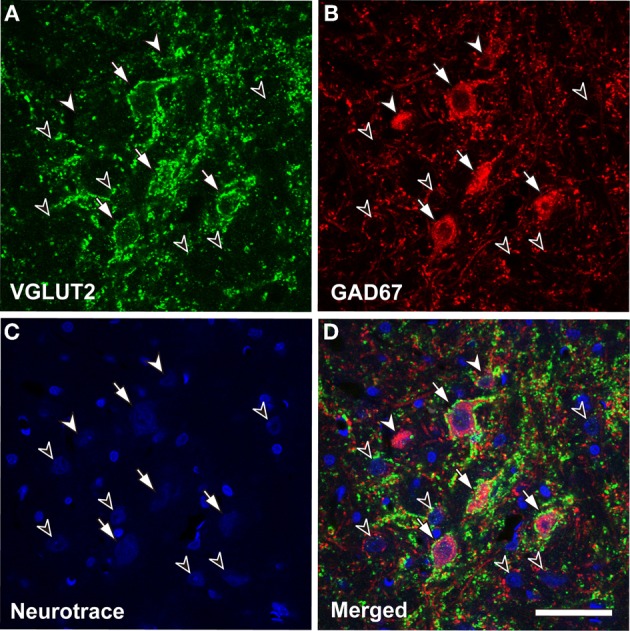
**Glutamatergic axosomatic terminals on large GABAegic (LG) neurons.** VGLUT2-immunopositive terminals (**A**, green) make dense axodendritic and axosomatic contacts (**A**–**D**, arrows) on GAD67-immunopositive (**B**, red) LG neurons. Smaller GAD67-positive neurons (**A**–**D**, SG, white arrowheads) and GAD67-negative cells (**A**–**D**, black arrowheads) do not receive VGLUT2 axosomatic terminals. Neurotrace Nissl Stain (blue) is shown in **C**. Bar: 50 μm.

The VGLUT2 axosomatic terminals are seen only on large GABAergic (LG) neurons, and they are absent on other cell types. Only neurons that were immunopositive for GAD67, a synthetic enzyme for GABA and a marker for GABAergic neurons, had VGLUT2 axosomatic endings (98.9% ± 0.34; mean ± S.D., *N* = 3; Figure 1). All LG neurons (diameter >16.5 μm) received VGLUT2 axosomatic endings, but small GABAegic (SG) neurons (diameter <10.7 μm) did not. The two types of GABAergic neurons overlapped at intermediate sizes. Nevertheless for simplicity, we will use the term LG to refer to GABAergic neurons with VGLUT2 axosomatic terminals and the term SG to refer to GABAergic neurons lacking those endings.

Samples of IC GABAergic neurons were collected with stereological methods (Ito et al., 2009), and the total population of LG and SG neurons was estimated (Table 1). There were 32,495 ± 4607 (mean ± S.D., *N* = 4) LG neurons in the IC out of 56,490 ± 7424 total GABAergic neurons. Thus, the ratio of LG to all GABAergic cells is 57.5% ± 1.5^1^. Since the IC is estimated to have 373,600 neurons (Kulesza et al., 2002), roughly 10% of IC neurons are the LG type. These results suggest that LG neurons are the largest group of GABAegic IC neurons and represent a substantial amount of the entire population of IC neurons.

**Table 1:** **Stereological estimates of GABAergic neurons in the IC (mean ± S.D., *N* = 4)**.

Estimated number of all GABAergic neurons	56,490 ± 7424
Estimated number of LG neurons	32,495 ± 4607
Estimated number of SG neurons	23,995 ± 3032
Estimated number of IC neurons (Kulesza et al., 2002)	373,600

### Distribution of LG neurons

LG neurons are found in all subdivisions of the IC, but the ratio of LG neurons to SG neurons differs between subnuclei. The proportion of LG to total GABAergic cells is higher in the ICC (73.2% ± 6.0^*^), lower in the cortices (DC: 51.4% ± 7.3^*^, LC: 47.7% ± 15.3^*^), and lowest (about 20%; calculated from non-stereological samples; Figure 5B of Ito et al., 2009) in the GABA modules (Chernock et al., 2004) of the LC. This suggests that LG neurons are more important for the ICC functions than cortical functions.

The density of each class of GABAergic neuron in the IC was calculated for each subdivision (Figure 5B of Ito et al., 2009) (Table 2). LG neurons have the highest density in the ICC; however, there was no significant difference in the density in most other subdivisions. The exception was layer 1 of the LC that has significantly fewer LG neurons than the ICC (*P* = 0.0012, Tukey’s multiple comparison test). In contrast, the density of SG neurons was especially high in the GABA modules but very low in layer 1 of the LC. In the other subdivisions, there was no significant difference in the density of SG neurons. Thus, there is little difference in the density of LG and SG neurons for most IC subdivisions. The exceptions are LC layer 1 and the GABA modules that may have a local circuit that differs from most of the IC. Indeed, LC layer 1 and the GABA modules have GABAergic neurons that are distinguished by their phasic responses to current injection (Ono et al., 2005), and both receive strong corticofugal input (Chernock et al., 2004; Winer, 2005). Since the other subdivisions, i.e., ICC, DC, and layer 2&3 of LC, have a similar density of LG and SG neurons, they are likely to share a common local circuit with minor differences.

**Table 2:** **Density of two classes of GABAergic neuron in IC subdivisions**.

**Subdivision**	**Density (counts/ mm^2^)**	
	**LG**	**SG**
Whole IC	29.5 ± 6.9	42.7 ± 8.8
ICC	54.5 ± 8.2^A^	50.6 ± 13.8^B^^H^
DC	30.9 ± 5.5	50.8 ± 12^C^^G^
LC layer 1	0.5 ± 0.8^A^	10.0 ± 6.1^D^^F^^G^^H^
LC layer 2&3	33.3 ± 9.7	62.4 ± 4.3^E^^F^
GABA modules	41.3 ± 32.3	164.5 ± 5.7^B^^C^^D^^E^

A P = 0.012;

**BCDEF:** P < 0.001;

GH P = 0.002, Tukey’s multiple comparison test.

In the ICC, where LG cells are most numerous, the distribution of LG and SG neurons should not be clustered if the types of GABAergic neurons are not associated with specific inputs (see below). To assess the randomness of the distribution, we calculated Ripley’s *L* function (Ripley, 1976) from the distribution of the two classes of GABAergic neurons in the ICC (Figure 5B of Ito et al., 2009, Figure 2A). The *L* function gives the mean number of neurons separated by a distance smaller than *h* per density standardized with *h*. If the neurons form clusters, the *L* function will be larger than the confidence limits, but the L function is smaller than the confidence limits when the cell bodies are homogeneously distributed. Within the confidence limits, the distribution of the cell bodies cannot be distinguished from a random distribution. When *h* is one or two times the diameter of the cell body, it suggests a local deviation of the distribution, but a larger *h* (several hundred micrometers) implies a global deviation in the distribution.

**Figure 2:**
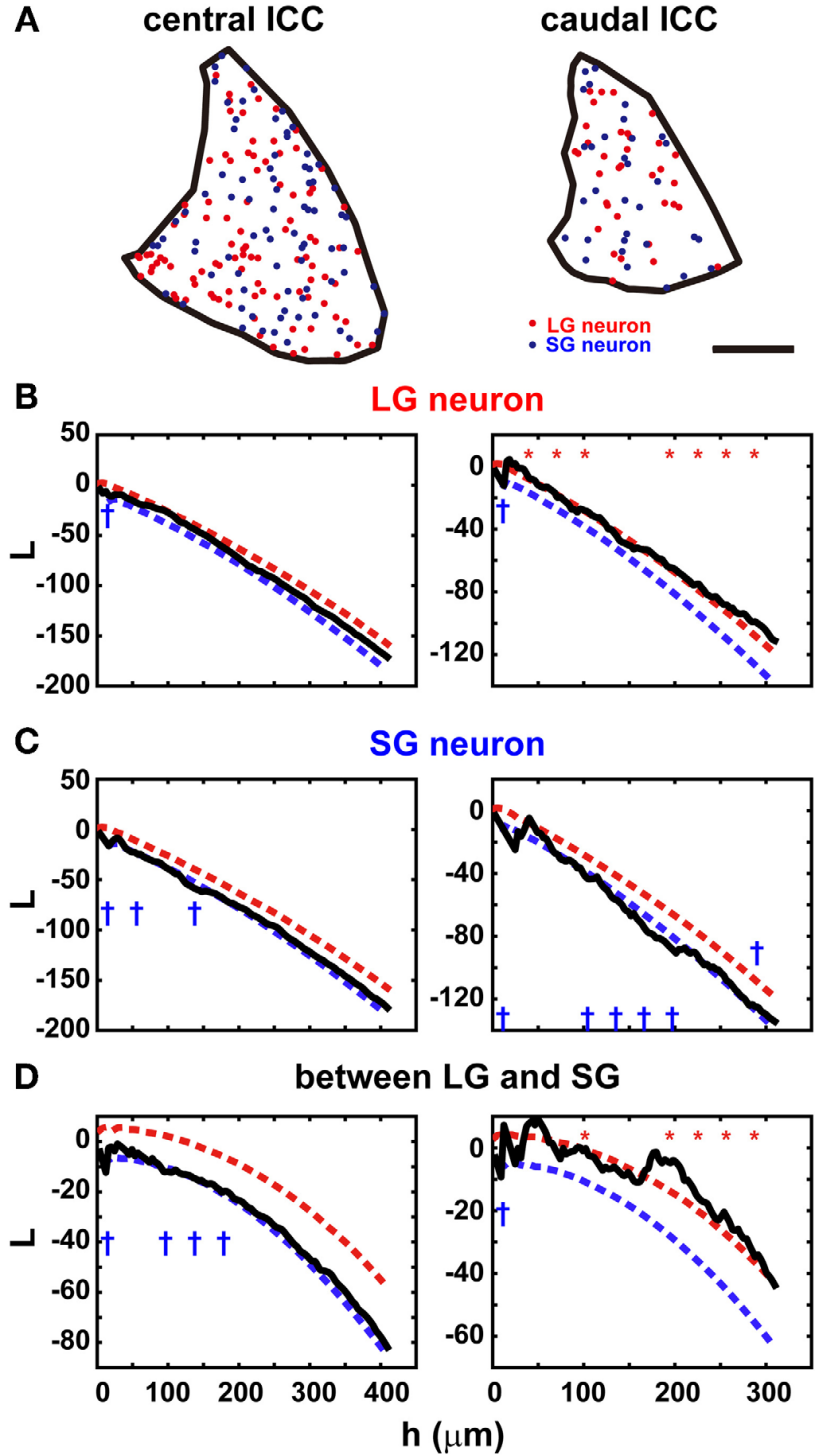
**Distribution in the ICC of two classes of GABAergic neurons. (A)** Location of the cell bodies of LG neurons (red) and SG neurons (blue) in the ICC (black border). Left: center of the ICC. Right: caudal ICC. Note the sparse and clustered distribution in the caudal ICC. Scale bar: 400 μm. **(B)** The *L* function of LG neurons in the central ICC (left) and caudal ICC (right). Red and blue dotted lines are upper and lower limits of the 95% confidence interval, respectively. In the central ICC, the function falls primarily within the confidence limits, while in the caudal ICC, the function is slightly larger than the confidence interval. At 10 points of *h* evenly spaced, asterisks and daggers are shown if the function is larger or smaller than the confidence interval, respectively. **(C)** The *L* function of SG neurons in the central ICC (left) and caudal ICC (right). In the central ICC, the function falls within the confidence limits in the larger values of *h*. When *h* is smaller, the function falls below the confidence limit. In the caudal ICC, the *L* function is significant for larger values of *h*. **(D)** reciprocal *L* functions for two classes of GABAergic neurons in the central ICC (left) and caudal ICC (right). In the central ICC, the function is significant for only the smaller *h*. In the caudal ICC, the function is significantly at larger values of *h*.

The distributions of both LG and SG neurons were estimated along the rostrocaudal axis of ICC. They were distributed randomly in the central part but less so in the rostral and caudal parts. In the central ICC, the distribution fell within the 95% confidence limits (left part of Figures 2B,C): we counted the number of times the *L* function exceeded the confidence limits at 10 evenly spaced points of *h* and found significant differences in only 0–3 points on average (Table 3). Even at the points where significance was detected, the difference between *L* functions and the confidence limit was very small (left part of Figures 2B,C). Therefore, we conclude that LG and SG GABAergic neurons are distributed randomly in the central ICC.

**Table 3:** **Mean number of points detecting significance in *L* functions**.

	**Rostral and caudal ICC (6 sections)**		**Central ICC (8 sections)**	
	**Larger**	**Smaller**	**Larger**	**Smaller**
LG neuron	6.3	2.7	1.4	1.1
SG neuron	2.2	3.5	0.3	2.9
Between LG and SG	4.0	2.8	0.8	2.3

For each L function, 10 points of h are examined for significance.

In contrast, in the rostral and caudal part of the ICC, *L* functions of LG neurons were significantly larger than the 95% confidence interval (right part of Figure 2B; 5/6 sections, *N* = 3) over a large range of *h* values, and this suggests a clustered distribution for LG neurons. For SG neurons, the *L* functions were smaller than the 95% confidence interval in three of six sections over a wide range of *h* values. This suggests a more uniform distribution for SG cells. Although there is a possible clustering of LG neurons in the rostral and caudal ICC, fewer LG neurons are found in these regions (32.2% ± 4.9 of all GABAegic neurons) than in the central ICC. Therefore, the majority of LG neurons did not make clusters, and this implies that LG neurons are randomly distributed and available equally to all inputs to the ICC.

The two classes of neurons appear to be distributed independently of each other in the ICC (Figure 2D). In the rostral and caudal ICC, *L* functions were significantly larger than the confidence interval over a large range of *h* values (4/6 sections). This implies the two classes of GABAergic neurons tend to cluster together. In contrast, in the central ICC the *L* functions were usually within the confidence levels (left part of Figure 2D). Thus, in the central ICC where the cells are most numerous, the LG and SG neurons are independently distributed.

## Inputs to the IC

It is clear that the major subdivisions of IC receive different inputs. A basic circuit for the IC should be composed of common components (Oliver, 2005). Within the subdivisions of the IC, there may be further regional subdivisions, e.g., functional zones, in which axons from a subset of afferent sources dominantly the input (Oliver et al., 2003; Cant and Benson, 2006; Loftus et al., 2010). The detailed organization of the inputs to the IC is beyond the scope of this review. However, the concept of a basic circuit suggests that within each subdivision or functional zone, there may be some common cell types with a common pattern of synaptic input. This is the aspect of inputs we wish to consider.

### Origins of excitatory inputs to the IC

It appears that all IC neurons receive axodendritic synapses from glutamatergic sources. These may come from a variety of sources depending on the subdivision of IC or functional zone (see above). Only the LG neuron has axosomatic glutamatergic synapses. What is the source of these inputs? As noted above, there appears to be different types of glutamatergic input. Only VGLUT1 and VGLUT2 are present in the adult IC, but there are three patterns of VGLUT expression in axonal terminals: VGLUT1 only, VGLUT2 only, and a colocalization of both (Ito et al., 2009). Any of these patterns may be seen in axodendritic terminals, but the axosomatic terminals on LG neurons were only positive for VGLUT2 (97.1% ± 1.8). In order to identify the origin of the VGLUT2 axosomatic endings, the expression of VGLUT1 and VGLUT2 mRNA in neurons that project to the IC was used since the VGLUT proteins are seldom seen in the cell body of neurons.

**Figure 3:**
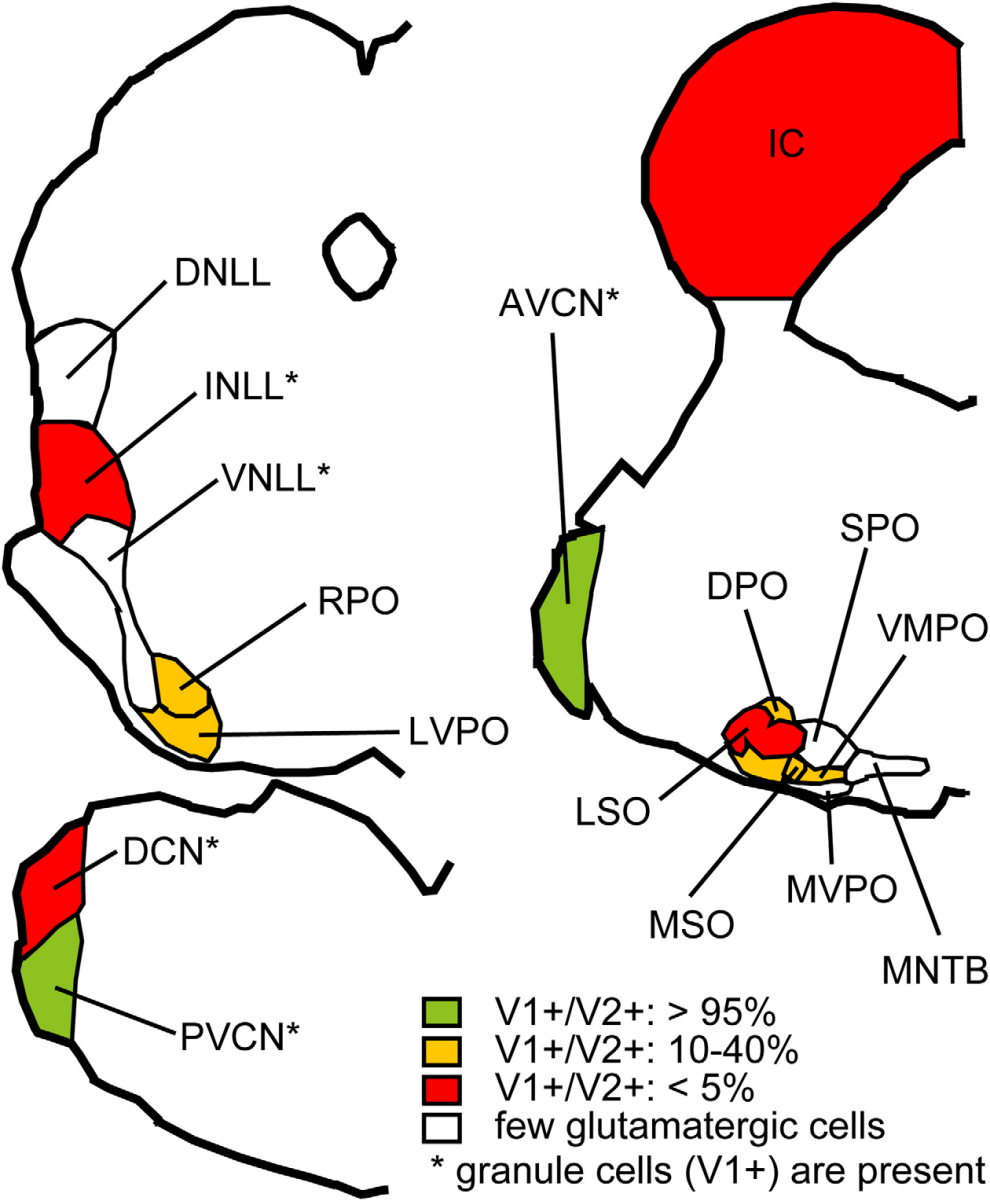
**Summary of VGLUT mRNA expression in the rat auditory brainstem.** Red: Nuclei where less than 5% of glutamatergic neurons express both VGLUT1 and VGLUT2 and other glutamatergic neurons express VGLUT2 only. Green: Nuclei where more than 95% of glutamatergic neurons express both VGLUT1 and VGLUT2. Orange: Nuclei where 10–40% of glutamatergic neurons express both VGLUT1 and VGLUT2, and other glutamatergic neurons express VGLUT2 only. White: Nuclei with almost no glutamatergic neurons. Note that granule cells, expressing VGLUT1 only, are found in nuclei with asterisks, and excluded from analysis. Abbreviations: DNLL, dorsal nucleus of the lateral lemniscus; AVCN, anteroventral cochlear nucleus; PVCN, posteroventral cochlear nucleus; MVPO, medioventral periolivary nucleus; SPO, superior paraolivary nucleus; MNTB, medeial nucleus of the trapezoid body. For other abbreviations, see text. Modified from Ito et al. (2011).

*In situ* hybridization for VGLUT1 and VGLUT2 was performed in the auditory brainstem of the rat and mouse (Ito et al., 2011). Gene expression for these transporters was found in most brainstem centers including in the IC, the intermediate nucleus of the lateral lemniscus (INLL), the dorsal and ventral cochlear nuclei (DCN and VCN), and the superior olivary complex (SOC) including the lateral superior olive (LSO), medial superior olive (MSO), rostral periolivary (RPO), dorsal periolivary (DPO), lateroventral periolivary (LVPO), and ventromedial periolivary nuclei (VMPO) (Figure 3). Neurons expressing genes for VGLUT were absent or very sparse in the dorsal and ventral nuclei of the lateral lemniscus (DNLL and VNLL), medial nucleus of the trapezoid body (MNTB), superior paraolivary nucleus, (SPO), and medioventral periolivary nucleus (MVPO).

Expression of VGLUT2 alone was the most common pattern, and a smaller number co-expressed VGLUT1 (Figure 3). Only a few cells expressed VGLUT1 alone, and these were confined to the granule cells in the cochlear nuclei, granule cells in the nuclei of the lateral lemniscus, and neurons in DPO and VMPO. The incidence of colocalization of VGLUT2 and VGLUT1 differed among nuclei. VGLUT2 was never colocalized in the IC. In the DCN, INLL, and LSO, co-expression of VGLUT1 and VGLUT2 was rare and in fewer than 5% of the glutamatergic neurons (Figure 3, red). In the DPO, LVPO, and MSO, more neurons co-expressed VGLUT1 (10–25%), and 30–40% of neurons in the RPO and VMPO co-expressed VGLUT1 (Figure 3, yellow). In the VCN, the vast majority of glutamatergic neurons (95%) expressed both VGLUT1 and VGLUT2 (Figure 3, green). Auditory cortex is also likely to be a source of VGLUT1 terminals since most cortical pyramidal neurons express only VGLUT1 (Fremeau et al., 2001; Herzog et al., 2001). Neurons with only VGLUT1 expression can be excluded from consideration as a source of the VGLUT2 axosomatic terminals on LG neurons.

### Putative sources of excitatory axosomatic inputs on LG neurons

**Figure 4:**
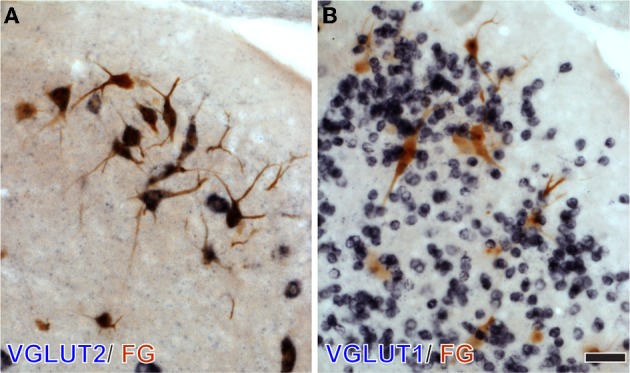
**DCN neurons projecting to the IC express VGLUT2 but not VGLUT1.** After an injection of Fluorogold (FG) into the IC, large DCN neurons, presumably fusiform and giant cells, show immunoreactivity for FG (brown). Their cell bodies are positive for VGLUT2 mRNA (dark blue in **A**), and negative for VGLUT1 mRNA **(B)** Note that numerous granule cells express VGLUT1 only. Bar: 40 μm.

**Figure 5:**
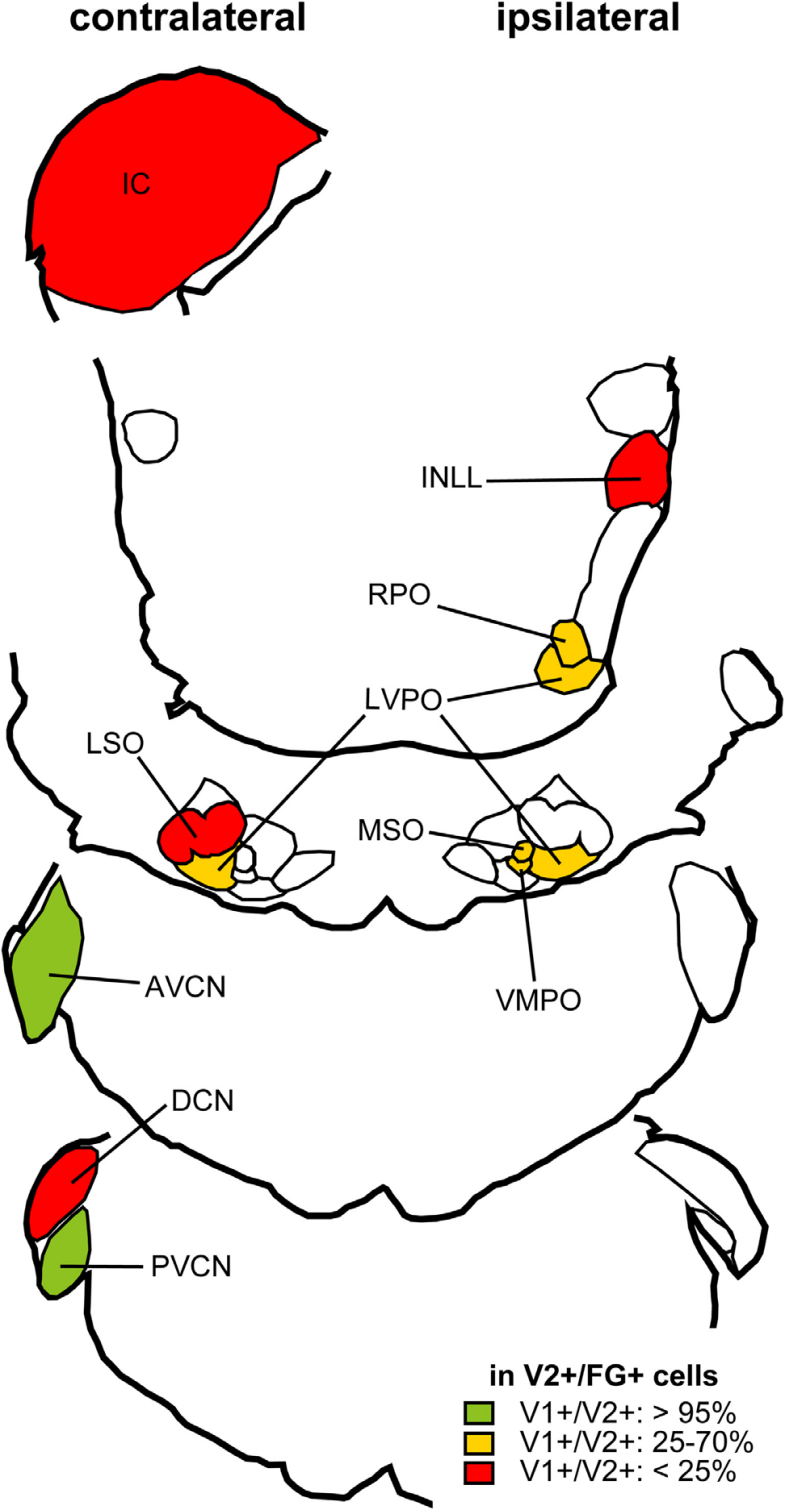
**Summary of distribution of retrogradely labeled glutamatergic cells after an injection of FG into the IC.** Percentage of neurons positive for FG and VGLUT2 that co-localize VGLUT1 calculated from three cases. Nuclei that have more than 10 mean cell count of cells positive for both FG and VGLUT2 are shown. Red: Brainstem regions where fewer than 25% of cells express VGLUT1. Green: Nuclei where more than 95% cells express VGLUT1. Orange: Nuclei where 25–70% of retrogradely labeled cells co-expressed VGLUT1. Modified from Ito and Oliver (2010).

To test whether specific VGLUT-expressing cells project to the IC, we performed *in situ* hybridization for VGLUT1 and VGLUT2 combined with retrograde tracing (Ito and Oliver, 2010). After an injection of Fluorogold (FG), a fluorescent retrograde tracer, into the IC, retrogradely labeled neurons were found throughout the auditory pathways and cortex. Four patterns of VGLUT gene expression in FG-positive cells were found; (1) VGLUT1 only, (2) VGLUT2 only (For example, fusiform cells of the DCN; Figure 4), (3) co-expressed VGLUT1&2, and (4) no VGLUT expression. FG-positive cells expressing VGLUT1 alone were found only in the auditory cortex. Nuclei that had a substantial number of FG-positive cells expressing VGLUT are shown in Figure 5,^2^. The most likely sources of the VGLUT2 axosomatic terminals on LG neurons (Figure 5, red) were the ipsilateral INLL, the contralateral LSO, the contralateral DCN, and the IC, since other nuclei have fewer neurons that express VGLUT2 only. These sources may also produce the numerous VGLUT2-positive terminals on dendrites in the IC.

## Outputs of the IC

### Tectothalamic inhibitory neurons

One of the main outputs of the IC is the auditory thalamus, the MGB. Both disc-shaped and stellate cells project to the MGB (Oliver, 1984; Oliver et al., 1991). Some of the tectothalamic neurons are GABAergic (Winer et al., 1996). GABA-immunopositive neurons were labeled were found in all subdivisions of the IC after retrograde tracing from the MGB. In cats, GABAegic neurons comprised 20% ± 9 of the tectothalamic neurons in the ICC (mean ± S.D., *N* = 5, Winer et al., 1996) and a slightly higher proportion in the cortices (LC: 20% ± 13, DC: 28% ± 12). In rats, the percentage was slightly higher (around 40% in the ICC and LC) except for the DC (20%, Peruzzi et al., 1997). These results suggest that a substantial amount of GABAergic tectothalamic neurons are found in all IC subdivisions and are the one of the output components of the IC’s basic circuit.

### LG neurons with axosomatic endings are tectothalamic cells

GABAergic axons in the brachium of the IC have a larger diameter than non-GABAergic axons (Saint Marie et al., 1997). Semithin cross sections of the brachium of the IC immunostained for GABA revealed that GABAergic fibers had larger diameters than non GABAergic axons, and the largest axons (diameter >4 μm) were exclusively GABA-immunopositive. The LG neurons are the most likely source for these LG axons. To determine whether LG neurons project to MGB, neurons in the IC were studied after retrograde labeling from the MGB (Ito et al., 2009). After injecting FG into the MGB, both LG and SG tectothalamic cells were identified by immunohistochemistry for GAD67, VGLUT2, and FG. The majority of the GABAergic tectothalamic neurons were LG neurons, and they encircled by VGLUT2-positive axosomatic endings (81.8% ± 12.4, 68.3% ± 7.6, and 75.1% ± 13.3 in ICC, DC, and LC, respectively). There were significantly fewer LG neurons in the GABA modules of the LC (40.3% ± 18.4) when compared to other subdivisions. Since the incidence of LG neurons was different between subdivisions, a “preference ratio” was calculated. The percentage of tectothalamic LG neurons to all tectothalamic GABAergic cells was divided by the percentage of LG neurons to all GABAergic neurons. In all subdivisions, the ratio was larger than 1.5 (1.56 in ICC, 1.87 in DC, 1.97 in LC, and 2.13 in GABA modules) and indicates that the LG neurons is the predominant GABAergic cell type in the projection from the IC to the MGB.

Although the majority of tectothalamic inhibitory neurons are the LG type, 20–30% of inhibitory projection was made by SG neurons. This suggests that there should be more variability in the IPSPs in the MGB. However, in brain slice experiments, the IPSPs had relatively uniform short latencies (Peruzzi et al., 1997) to suggest that inhibitory tectothalamic inputs to MGB are mainly LG inputs. Since relatively large injections of FG were made in the MGB, neurons sending axons to structures adjacent to the ventral division of the MGB could have been labeled. It is possible that some SG neurons project to non-lemniscal auditory thalamic nuclei or non-auditory nuclei in the neighborhood of the MGB such as the suprageniculate nucleus and posterior intralaminar nucleus.

### Targets of tectothalamic inhibitory cells

Not all MGB neurons receive inhibitory inputs from the IC. Bartlett and Smith (2002) recorded responses of MGB neurons during stimulation of the brachium of the IC. MGB neurons that received a smaller amplitude and longer latency excitatory input were the recipient of tectothalamic inhibitory projections, while neurons with larger amplitude and shorter latency excitatory inputs were not. The former neurons are likely to be innervated by small axon terminals, and the latter are likely to be innervated by large terminals. This data suggests the presence of at least two types of tectothalamic excitatory neurons. One of these excitatory tectothalamic excitatory neurons may share the same target MGB neurons with the inhibitory tectothalamic neuron.

A second study mapped the origin of the inputs from the IC onto a single MGB neuron (Lee and Sherman, 2010). Not all MGB neurons received the tectothalamic inhibitory input. Inhibitory and excitatory tectothalamic neurons that share the same postsynaptic MGB neuronwere always topographically segregated. This suggests a large degree of tectothalamic convergence. Both studies demonstrated that both tufted and stellate neuron types in the MGB may receive the tectothalamic inhibitory input, while only tufted neurons sometimes lack the tectothalamic inhibitory input (Bartlett et al., 2000; Lee and Sherman, 2010).

## Basic circuit of the IC

**Figure 6:**
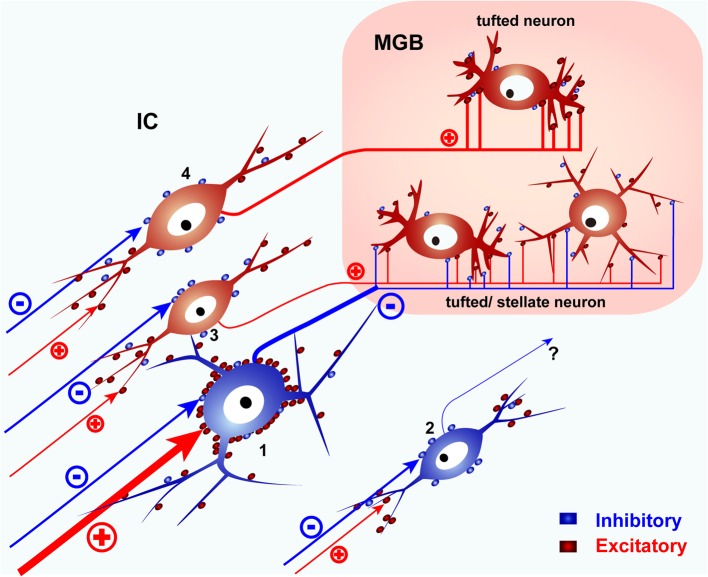
**A schematic diagram of the basic IC circuit.** LG neurons (1) receive strong excitatory inputs on their somata, send their axons to the MGB, and presumably inhibit tufted or stellate neurons in the MGB. SG neurons (2) do not target MGB. Glutamatergic neurons (3, 4) project to the MGB but lack the dense VGLUT2 axosomatic inputs. Glutamatergic neurons with small terminals (3) co-innervate tufted or stellate neurons with LG neurons. Other tufted neurons are innervated by glutamatergic neurons with large terminals (4) and do not receive inputs from LG neurons. SG and glutamatergic neurons receive most of their excitatory inputs on their dendrites. Red puncta indicate excitatory glutamatergic terminals. Blue puncta indicate inhibitory (GABAergic and glycinergic) terminals. Modified from Ito et al. (2009).

Since LG neurons are found in all IC subdivisions and distributed randomly in the ICC (Figure 2), they are not likely to be embedded in specific pathways, and they may have a common function in the ICC and other parts of the IC. Accordingly, the basic circuit of the IC must contain at least three types of neurons that project from the IC to the MGB, the two types of glutamatergic neurons which make large and small EPSPs on the MGB cells and the LG neuron (Figure 6). SG neurons are less likely to project to MGB and may have other targets like the contralateral IC. With two types of glutamatergic input and one type of GABAergic input, the information transmitted by this circuit to the MGB will depend on how and when these different inputs are activated.

Since LG neurons are the largest IC neurons, they might be expected to be sluggish and have a longer latency than smaller IC cells. For example, they might have greater membrane capacitance to overcome before they fire. It is possible that the dense VGLUT2 axosomatic terminals may help the LG neurons overcome this capacitance and fire more quickly despite their large size. A recent *in vivo* study investigated the properties of visually identified neurons of different size in the dorsal IC cortex of mouse (Geis and Borst, 2011). The largest cells had a low input resistance. When responses were evoked by sound, the largest cells had short latency excitatory synaptic responses, and they fired action potentials with a short latency. The sound evoked excitatory input was often followed by longer latency inhibitory postsynaptic potentials. These data are consistent with the LG neuron as a source of short latency inhibitory input to the MGB.

Since an LG neuron is also likely to have an axon with a large diameter in the brachium of the IC (Saint Marie et al., 1997), its IPSP actually may arrive at the MGB before the EPSP from the glutamatergic IC neuron assuming these two IC neurons fire simultaneously. This was the finding after electrical stimulation of the brachium of the IC (Peruzzi et al., 1997). Therefore, the relative timing of action potentials in LG neurons and glutamatergic tectothalamic IC neurons will determine the signals arriving at the MGB.

If LG neurons are driven more by local glutamatergic IC sources than ascending afferent sources, the IPSPs produced by LG neurons may reach the MGB simultaneously or later than the EPSPs from glutamatergic IC neurons. A local or recurrent excitation of the LG neuron might erase the 2 ms lead time of the IPSP over the EPSP after BIC shock (Peruzzi et al., 1997). In that case, the MGB neuron will receive almost simultaneous excitatory and inhibitory inputs, comparable to the triad synapses formed by glutamatergic inputs from IC and local GABAergic dendrites in the cat (Morest, 1971, 1975). In that synaptic arrangement, excitation is followed by very short latency inhibition.

Since rodents lack local GABAergic neurons in the MGB (Ito et al., 2011), one may suspect that the LG neurons are a compensatory mechanism in the rodent. However, LG neurons have been observed in Japanese macaque, marmoset, and rabbit where GABAergic neurons are present in the MGB (unpublished observations, Ito and Takada, 2011). Thus, the LG tectothalamic neurons in the IC may not be a rodent specialization but rather a fundamental neuron type in the IC. That may differentiate its role from that of the MGB triad synapse. LG neurons may receive more convergent input than the MGB triad. The LG neurons are the largest neurons in the IC, and they are likely to have a stellate dendritic field that promotes additional convergence especially in the DC and LC (Oliver et al., 1994). Additional convergence may be seen in the MGB (Lee and Sherman, 2010). In contrast, the triad synapse may have less convergence since the same IC afferent drives both excitatory afferent and the dendro-dendritic GABAergic synapse.

If axosomatic terminals on LG neurons arise from multiple sources, the arrival of the IPSP on the MGB neurons may be variable and context dependant. Both faster and slower EPSPs will be possible. We do not know yet how many VGLUT2 axosomatic terminals must be active to fire a LG neuron. Calyx- or endbulb-type synapses have not been found in the IC, so the VGLUT2 axosomatic synapses are not from a single axon. This suggests that a highly synchronized excitatory input to the LG neuron is not as likely as a more asynchronous event. In the latter case, some acoustic stimuli may activate the excitatory inputs on a LG neuron more than other stimuli. Thus, the selective firing of excitatory inputs to the LG neuron might filter the inhibition received by the auditory thalamus.

## Materials and methods

### Statistical analysis

#### Distribution of GABAergic cells

Data obtained from three Long–Evans rats in the previous study (Ito et al., 2009) were used. Twenty-μm-thick IC coronal sections were collected, and every 24th section was immunostained for GAD67 and VGLUT2, and then counterstained with Neurotrace 510 (Invitrogen, Grand Island, NY). Photomicrographs of the whole IC were taken by a laser scanning confocal microscope, and montage images of the whole IC were made from four IC sections at interval of 480 μm.

#### L function

To measure both local and global dispersion of GABAergic neurons in the ICC, we counted the number of neurons located within a distance of *h* from other neurons. Ripley’s *K* function (Ripley, 1976) is calculated as:

$$K=\frac{\sum_{i\neq j}I(d_{ij}<h)}{n\lambda }$$

where *I* is the number of neurons, *d*_*ij*_ is the distance between *i* th and *j* th neurons in a set of *n* neurons, and λ is the density of neurons. For data analysis, the function is standardized with *h* in a following manner:

$$L=\sqrt{K/\pi }-h$$

#### Monte-Carlo simulation

To test whether the obtained *L* functions show random, uniform, or concentrated distribution, a 95% confidence interval of given ICC region was calculated by Monte-Carlo simulation in the following manner. First, neurons were randomly re-distributed, and the *L* function was calculated. The process was repeated 10,000 times, and the distribution of the *L* function of random distribution was obtained. If the original *L* function was outside of the 95% confidence interval at given *h*, the distribution is not likely to be random at the range *h*. If the *L* function is smaller than the confidence interval, neurons are more uniformly distributed. If it is larger, they are more concentrated. At 10 points of *h* evenly separated, significant difference between *L* function and the confidence interval was examined, and mean counts of significance were calculated (Table 3). The *L* functions and confidence intervals were calculated with custom-made scripts on MATLAB Statistics Toolbox (version: R14; Mathworks, Natick, MA).

#### Stereological estimate of GABAergic cells

Data obtained from four Long Evans rats in the previous study (Ito et al., 2009) were used. Twenty-μm-thick IC coronal sections were collected, and every 12th section was immunostained for GAD67 and VGLUT2, and counterstained with Neurotrace 510. IC sections were divided into a 500 × 500 μm grid, with a 57.1 × 57.1 × 20 μm box in the center as a sampling frame. If nucleoli of GAD67-positive cells were found inside the box, images of the cells were taken, and categorized as LG or SG by the presence or absence of dense axosomatic VGLUT2-positive endings. After acquiring stereological samples, low-magnification montage images of the IC were taken to locate the sampling frames and to measure the size of the IC. The density of GABAergic neurons was calculated by dividing the total number of GABAergic neurons by number of the sampling frames and volume of a sampling frame. Total volume of the IC was estimated by linear interpolation of the volume of every 12th section. Estimated number of GABAergic neurons was calculated by multiplying the density with total volume of the IC.

### Conflict of interest statement

The authors declare that the research was conducted in the absence of any commercial or financial relationships that could be construed as a potential conflict of interest.

## Footnotes

^1^The ratio is slightly different from our previous result, which was obtained from montage of whole IC image (Figure 5B of Ito et al., 2009) but was not stereological. The new ratio was calculated from stereological samples (Figure 4 of Ito et al., 2009).

^2^For the scope of this review, we ignored FG-positive cells not expressing VGLUT from the original data of Ito and Oliver (2010).
